# The Effect of Including an Opt-Out Option in Discrete Choice Experiments

**DOI:** 10.1371/journal.pone.0111805

**Published:** 2014-11-03

**Authors:** Jorien Veldwijk, Mattijs S. Lambooij, Esther W. de Bekker-Grob, Henriëtte A. Smit, G. Ardine de Wit

**Affiliations:** 1 Centre for Nutrition, Prevention and Health Services, National Institute for Public Health and the Environment, Bilthoven, The Netherlands; 2 Julius Center for Health Sciences and Primary Care, University Medical Center Utrecht, Utrecht, The Netherlands; 3 Department of Public Health, Erasmus MC, University Medical Center, Rotterdam, The Netherlands; University of Ottawa, Canada

## Abstract

**Objective:**

to determine to what extent the inclusion of an opt-out option in a DCE may have an effect on choice behaviour and therefore might influence the attribute level estimates, the relative importance of the attributes and calculated trade-offs.

**Methods:**

781 Dutch Type 2 Diabetes Mellitus patients completed a questionnaire containing nine choice tasks with an opt-out option and nice forced choice tasks. Mixed-logit models were used to estimate the relative importance of the five lifestyle program related attributes that were included. Willingness to pay (WTP) values were calculated and it was tested whether results differed between respondents who answered the choice tasks with an opt-out option in the first or second part of the questionnaire.

**Results:**

21.4% of the respondents always opted out. Respondents who were given the opt-out option in the first part of the questionnaire as well as lower educated respondents significantly more often opted out. For both the forced and unforced choice model, different attributes showed significant estimates, the relative importance of the attributes was equal. However, due to differences in relative importance weights, the WTP values for the PA schedule differed significantly between both datasets.

**Conclusions:**

Results show differences in opting out based on the location of the opt-out option and respondents' educational level; this resulted in small differences between the forced and unforced choice model. Since respondents seem to learn from answering forced choice tasks, a dual response design might result in higher data quality compared to offering a direct opt-out option. Future research should empirically explore how choice sets should be presented to make them as easy and less complex as possible in order to reduce the proportion of respondents that opts-out due to choice task complexity. Moreover, future research should debrief respondents to examine the reasons for choosing the opt-out alternative.

## Background

There seems to be consensus regarding the inclusion of an opt-out option in Discrete Choice Experiments (DCEs) that aim to determine the potential participation in an elective program as such an option is more in accordance with the respondent's choice options in real life [1]–[4]. Moreover, when estimating the potential number of participants in any program, insight into the percentage of the target population that does not wish to participate in such a program is necessary. However, if individual preferences are measured to determine which components define the most preferred program or treatment, the inclusion of an opt-out option might not be a necessity but rather a threat to efficiency. Until now, the choice to include an opt-out option is determined by the objective of the DCE in the first place. Nevertheless, very little empirical evidence exists on the issue whether, and to what extent the inclusion of opt-out options in DCEs effect choice behavior of respondents. Which may therefore influence the precision of the estimates of attributes, the relative importance of the attributes, trade-offs (e.g., willingness to pay) calculated based on these estimates and thereby the conclusions that will be drawn from a DCE.

Most DCEs in health economics are rooted in the Random Utility Theory (RUT) [3], [5]–[7]. This theory assumes that respondents choose rationally and will select the scenario that generates the highest personal utility, that is, respondents will only select the opt-out option if none of the presented scenarios in that specific choice task is more attractive than the opt-out option [5], [8]. Additional research shows that from this perspective, forcing respondents to make a choice induces bias, as they would not always make that same choice in real life [3], [9], [10]. In such a forced-choice situation, people who would rather choose to opt-out, tend to randomly select either scenario from a choice task or select the most safe/least extreme scenario [9]–[12]. As a consequence, the standard error of the attribute estimates will increase while the external validity decreases [9], [10]. In summary, based on the RUT, an opt-out option can always be included, if this is accordance with the respondent's real-life decision context.

However, in practice, other motives than achieving the highest personal utility may be more important when people make their decisions [8]–[22]. This resulted in the hypothesis that only very few respondents act solely according to the assumptions of the RUT when choosing the opt-out option. Some individuals are more prone to choose the opt-out situation even before they actually evaluate the different situations in a choice task. Baron and Ritov (1992) argued that individuals choose the opt-out alternative to protect themselves from poor choices, as negative outcomes based on taking action (choosing) are perceived as worse compared to negative outcomes due to inactivity (not choosing) [19]. This finding was confirmed by many others [13], [17], [18], among which a theory by Luce and colleagues who suggest that if people decide to make a choice, the tendency to choose to opt-out increases as the trade-off becomes more difficult and the decision at hand is emotion-laden [12], [16]. This indicates that people choose to opt-out to avoid making difficult trade-offs [12], [16]. Research by Dahr and colleagues (1997 and 2003) showed that choice task complexity (i.e., large number of choice situations per choice task or comparable choice situations with respect to their attractiveness) results in more opting out [9], [11]. In summary, it seems plausible that respondents choose the opt-out option more often if they have to decide about a complex emotion-laden topic, if choice tasks are difficult, if scenarios are complex and if none of the scenarios is clearly superior. This way, respondents minimize their effort and reduce internal conflict induced by (negative) decision making.

The above is of special interest within the public health setting. Decisions about personal health, public health and prevention are by definition complex and difficult and not part of an individual's everyday life decisions [23]–[25]. Because DCE data are most often analysed according to the assumptions of the RUT, it can be discussed to what extent DCE results will be biased when respondents choose to opt-out as a result of reasons described above and not based on perceived personal utility. Until now, there is no empirical evidence on the effects of including an opt-out option on choice behaviour and the results of a health-related DCE. Therefore, the aim of this study is to investigate to what extent including an opt-out option in a DCE influences choice behaviour and thereby affects the attribute level estimates, relative importance of the attributes, calculated trade-offs, and thereby the conclusions drawn for this DCE.

## Methods

### Participants and recruitment

This study included participants diagnosed with type 2 diabetes mellitus (DM2), who were 35–65 years of age and who were not suffering from any serious complications due to their DM2. Participants were contacted via their diabetes care group. Within the Netherlands, diabetes care of all diagnosed DM2 patients is arranged in care groups, which are legal entities formed by multiple health care providers centered around general practitioners [26]. All Dutch care groups (n = 94) were categorized by the province of the Netherlands in which they are located. Per province, one care group was randomly selected and contacted, until five care groups agreed to participate. These five care groups distributed 2,500 questionnaires in total, among all the eligible DM2 patients who were registered at these care groups. The Dutch National Ethics Board (Central Committee on Research involving Human Subjects) concluded that formal testing by a medical ethical committee was not necessary, as T2DM patients were only required to complete an anonymous questionnaire once, which is in accordance with the guidelines laid down in the Declaration of Helsinki.

### Derived attributes and levels

Based on previously published literature on barriers and facilitators to participate in a lifestyle program among DM2 patients [27]–[37], interviews with experts (n = 3) and four focus group interviews with DM2 patients (total n = 24), five attributes with each three levels were selected for the current DCE. These included: menu schedule, physical activity (PA) schedule, consultation structure, expected outcome, and out-of-pocket costs.

A menu schedule and a PA schedule are plans that will be developed by the participants in the program together with a lifestyle coach. These plans describe the aims of the participants with respect to improvements in their diet and PA behavior. A flexible schedule is a schedule that is based mostly on the participants own initiatives and ideas. A general schedule is a schedule that includes general information on either a healthy diet or PA and provides example recipes or exercises. An elaborate schedule comprises a tailored schedule that is prepared mostly by the lifestyle coach. Consultation structure describes the composition of the consultations with the coach (i.e., individual or in groups of 5 or 10 other patients). These are the consultations during which the participants develop their menu plan and PA schedule, and during which they discuss their progress. The expected outcome is meant to describe the results with respect to weight loss (0, 5 or 10 kilograms) and physical fitness, which the respondent can expect to achieve after completing the lifestyle program. Finally, as the costs of participating in a lifestyle program are not part of the participant's health insurance in general, the participant will have to pay for (part) of the program out-of-pocket. These costs can amount to either €75, €150 or €225 per year.

### Experimental design

The scenarios in the DCE are constructed by combining different levels of each included attribute. The experiment comprises an unlabeled (generic) design with respect to the lifestyle program options. NGene 1.1 software (ChoiceMetrics, 2011) was used to create a D-efficient design for the current study. The software was instructed to create a design with two blocks using a panel-mixed-multinomial model with all beta-priors set at zero. It was assumed that there would be no interaction between attributes, while level balance, utility balance and minimal overlap between attribute levels were optimized [38], [39].

Finally, the design consisted of a sample of nine choice tasks per block (18 unique choice tasks in total). Within this design, each choice task contained two lifestyle program scenarios. To compare the possible differences in decision making when respondents are forced to make a choice or are offered an opt-out option (unforced choice option) and to obtain insight into the possible influence of the location of the opt-out option within the questionnaire [13], a within-sample design using four versions of the questionnaire was developed. This implies that version 1 and 2 of the questionnaire included the nine choice tasks of block 1 and version 3 and 4 included the nine choice tasks from block 2. Version 1 and 3 first offered the opt-out option and then forced respondents to choose, whereas version 2 and 4 started with forced choice tasks followed by choice tasks with an opt-out option (Table 1). To adjust for bias induced by the question order, the order of the choice tasks per version of the questionnaire was randomized. The opt-out option was included in the choice tasks as a third scenario, to prevent respondents from interpreting the opt-out option in different ways, all attributes were explicitly set to zero or ‘none’ in this scenario. Eventually, every respondent was asked to answer 18 choice tasks of which nine with the opt-out alternative and nine without.

**Table 1:** Overview of the content of every version of the questionnaire.

Questionnaire	Block	First nine choice tasks	Second nine choice tasks
Version 1	1	Including opt out	Excluding opt out
Version 2	1	Excluding opt out	Including opt out
Version 3	2	Including opt out	Excluding opt out
Version 4	2	Excluding opt out	Including opt out

### Pilot test

The questionnaire was pilot tested among a subgroup (n = 20) of the study population to ensure that the wording used in the questionnaire was correct and understood by the target population [40], [41]. During the pilot phase there was specific attention for the issue of interpretation of the opt-out option. Most of the pilot tests were distributed by means of postal questionnaires, respondents were asked to mark every question or answering category that they did not understand or found hard to grasp and they were asked to provide suggestions for improvement. Moreover, three think-aloud pilot tests were conducted to obtain more insight into the respondent's approach when answering the choice tasks. No changes in the attributes and/or levels were deemed necessary based on the results of this pilot study. Power/sample size calculations were performed based (partly) on the retrieved pilot data, to check a posteriori how large the sample size should be to find significant differences for each attribute at a 5% level in the final DCE [38], [42].

### Questionnaire

The questionnaire contained two parts. The first section of the questionnaire consisted of 28 questions about the respondent's demographic/background characteristics and on the patient's opinion with respect to lifestyle programs in general, accompanied by the EuroQol-5D health status questionnaire [43]. The second part was the actual DCE, which started with a detailed description of the attributes and levels and gave comprehensive guidance on how to answer the choice tasks provided. Every choice task started with the question: ‘Imagine that your general practitioner advises you to participate in a lifestyle program for one year, which program would you prefer: the program in situation 1 or situation 2?’ The following sentence was added to the above question in the choice tasks that included an opt-out option: ‘If you prefer not to participate in either of the situations, you can tick the box ‘none’.’

### Statistical analyses

According to the RUT, perceived utility (U) of a person ‘n’ in choice situation ‘j’ is estimated by the sum of the systematic utility component (V) (i.e., the mean utility of the target population concerning a specific topic including the same attributes and levels) and the random error term (ε) (i.e., the deviation of the utility of every single person ‘n’ compared to the mean utility) (equation 1.1) [5], [6], [44].
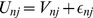
(1.1)


Based on the data retrieved by a DCE, the systematic utility component (V) of equation 1.1 can be estimated. This was estimated separately for the forced-choice data and the data that included an opt-out option. All analyses were conducted using mixed- logit (MIXL) models, to take preference heterogeneity into account. For the forced-choice data, the attribute estimates were estimated using equation 1.2 & 1.3.
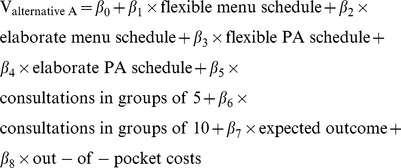
(1.2)

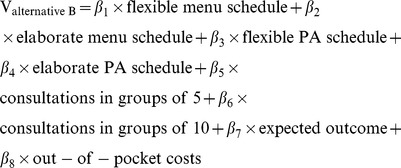
(1.3)β_0_ represents the alternative specific constant, while β_1_–β_8_ are the attribute estimates that indicate their relative importance. All included attributes were tested for linearity, the attributes that appeared not to be linear were effects coded (i.e., menu schedule, PA schedule and consult structure). In contrast to dummy coding, effects coding enables one to compare the estimates of all attributes despite their categorization into non-linear levels, because the effects are uncorrelated with the intercept [6], [45]. This coding procedure codes the reference category −1, therefore the sum of the effect coded attributes is always 0. The coefficient for the reference category is therefore −1*(βeffect code 1+βeffect code 2).

After comparing the model fits (based on AIC, BIC and Chi-square) of different models including different (sets of) random parameters, two parameters were set at random for the final analysis: the constant and the attribute expressing the expected outcome of the lifestyle program. In addition, different distributions of the random parameters were tested and based on the model fit results, both random parameters were included with a normal distribution. It was expected that especially the constant and the outcome attribute would show high preference heterogeneity among the respondents, due to large differences in general opinions concerning lifestyle programs and the variation in Body Mass Index (BMI) among the respondents.

The attribute estimates for the data that included the opt-out option was retrieved via equations 1.4–1.6. The β-values in this equation are to be interpreted as explained above for the forced-choice model, except for the constant term β0. Within this equation, β0 represents an alternative specific constant for both A and B, as opposed to the opt-out. The systematic utility of both A and B are modelled using the same constant term because the separate alternative specific constants for scenario A and B did not significantly differ from each other (based on the Wald test statistic).
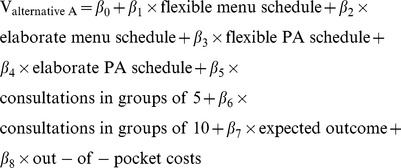
(1.4)

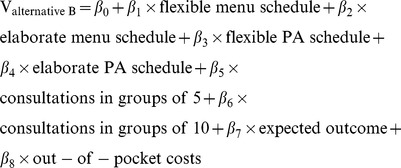
(1.5)

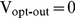
(1.6)


Choice consistency of the study population was checked [46]–[49], as every respondent answered every choice task twice (once they were forced to choose and once they were offered an opt-out option). Respondents satisfied the consistency measure if they chose the same option for every choice task in the first and second part of the questionnaire. Respondents who chose to opt-out were automatically marked consistent.

Differences in opting out between respondents with a lower (i.e., primary education or lower secondary education) and higher (i.e., all other levels) were determined using independent sample t-tests.

Marginal willingness to pay (WTP) values were determined for all statistically significant attribute estimates of the main analysis. These results can be compared directly between the forced-choice DCE and the DCE including the opt-out option. In order to calculate the patient's WTP, the negative of the out-of-pocket attribute was used as a measure of the marginal utility of money. The ratio of either attribute estimate with this negative of the out-of-pocket attribute was calculated to estimate the patient's WTP concerning that specific attribute [3], [50].

NLogit 5.0 (Econometric Software, New York) was used to construct the models that were estimated within this study. Results were considered statistically significant when p<0.05.

## Results

### Study population

Of all 2,500 distributed questionnaires, a total number of 781 (31.2%) questionnaires were returned and included in the analysis. The demographic and disease-specific characteristics of the study population are summarized in table 2. Respondents were aged 57.8 years on average and mainly Dutch (92.7%). Approximately half of the population was male (55.1%) and most respondents had an intermediate educational level (48.6%). With regard to the disease status of the respondents, they reported to have been diagnosed with DM2 on average 6.5 years ago and 79.7% used some form of medication. The mean BMI was 29.5 kg/m^2^ and the mean HbA_1c_ was 49.1 mmol/mol (target value for DM2 patients is <53 mmol/mol). The majority reported no complications due to their DM2 (75.6%) and the mean EQ-5D score was relatively high with 0.91. There were no clinically relevant differences in these demographic and disease-related variables between the respondents who completed the different versions of the questionnaire.

**Table 2:** General description of the study population (N = 781).

		Mean (SD)	Percentage
Age		57.8 (6.27)	
Gender	Male		55.1
Highest attained educational level	Low		31.2
	Medium		48.6
	High		20.2
Ethnicity	Dutch		92.7
Duration of diabetes (years)		6.5 (5.97)	
No complications			75.6
Medication	None		20.3
	Oral glucose lowering medication		66.7
	Insulin		4.0
	Both		9.0
BMI (kg/m^2^)		29.5 (5.19)	
HbA_1c_ (mmol/mol)		49.1 (14.0)	
EQ5d score		0.91 (0.19)	

### Choice behavior

With respect to the raw choice percentages, on average over all choice sets, 54% of the respondents were willing to participate in a lifestyle program when the option to opt-out was offered.

Respondents did not have a strong tendency to choose either option A or B. This was expected as A and B were unlabeled and therefore did not have specific characteristics which would make one of them more attractive within every choice tasks for the same reason (e.g., A was not always cheaper than B or the other way around).

The percentages of respondents choosing option A and B both dropped when the opt-out option was included. However, in 13 of the 18 choice tasks the difference in percentage of individuals moving from option A to the opt-out option and the individuals moving from option B to the opt-out option was more than 5%.

**Figure 1:**
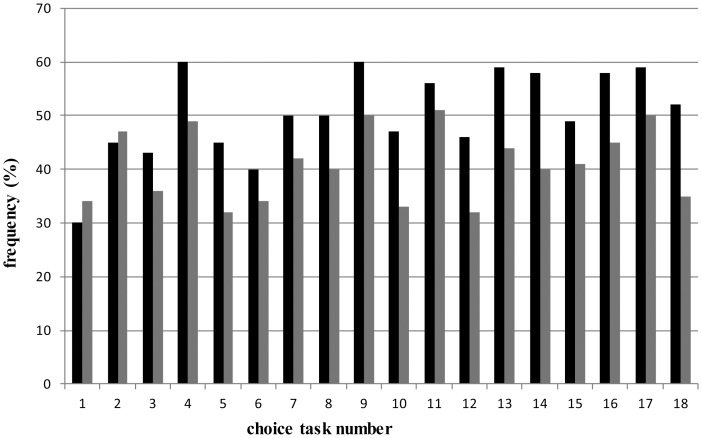
Proportion choosing to opt-out per choice task, stratified by respondents that started with the unforced choices (black) and respondent that were offered the opt-out in the second part of the questionnaire (grey).

In total 21.6% of the respondents always chose to opt-out, while 22.8% never chose to opt-out within the nine unforced choice sets. Respondents with a lower educational level significantly more often chose to opt out compared to respondents with a higher educational level (t = 2.31; P<.05). Except for choice task one and two, the frequency of choosing the opt-out option was significantly higher among respondents who first had the option to choose to opt-out and then were forced to make a choice, compared to respondents who first were forced to make a choice and later were able to choose the opt-out option (t = −2.94; P<.05) (Figure 1).

### Choice consistency

With respect to the consistency of the respondents when making their choices, overall 83.4% of the population answered consistently on all but one choice task. In total, 65.2% answered consistently on all choice tasks, while only 0.2% was consistent in six out of nine choice tasks (this was the lowest consistency score). The consistency measure was 93% on average per choice task.

### Attribute estimates and relative importance

Within both the forced and unforced model, respondents did not have any specific preferences with respect to a menu schedule (Table 3). They reported a strong dislike for a flexible PA schedule, compared to a more general schedule. An elaborate PA schedule was preferred over a general schedule only in the opt-out model. A consultation structure in which the respondents would work individually was preferred over consultations organized in groups with 10 other patients. Consultations in groups of five were preferred over groups of 10 only in the forced-choice model. In both models, respondents were more likely to choose a lifestyle program that was expected to result in higher weight loss and programs with the lowest out-of-pocket costs. Both models showed that out-of-pockets costs (per €100) were the most important attribute followed by expected outcome (per 10 kg of weight loss) consultation structure and PA schedule. Both models show significant (p<0.01) preference heterogeneity among respondents concerning the attribute that reflects the expected outcome of the lifestyle program in terms of weight loss, as was shown by the statistically significant standard deviation of this attribute in both datasets.

**Table 3:** Attribute estimates (standard errors) of the MIXL model.

		Forced choice model		Opt-out model	
Attribute		Beta value	SE	Beta value	SE
Constant	Mean	−0.11***	0.04	−0.30***	0.11
	Standard deviation	0.59	0.38	2.71***	0.52
Menu schedule	Flexible	−0.06	0.06	0.02	0.05
	General (ref)	0.10	0.12	0.02	0.04
	Elaborate	−0.04	0.04	−0.04	0.04
Physical activity schedule	Flexible	−0.25***	0.05	−0.14***	0.04
	General (ref)	0.18	0.07	0.01	0.05
	Elaborate	0.08	0.05	0.13***	0.04
Consultation structure	Individual (ref)	0.50***	0.06	0.51***	0.06
	Groups of 5	0.10**	0.05	0.02	0.04
	Groups of 10	−0.60***	0.08	−0.53***	0.06
Expected outcome (10 kg)	Mean	0.75***	0.13	0.76***	0.11
	Standard deviation	2.82***	0.59	1.63***	0.36
Out-of-pocket costs (€100)		−0.97***	0.10	−0.78***	0.06

**Significant at p<.05;

***significant at p<.001.

Location was included as a covariate in the analysis and turned out to be statistically significant (p<0.001), indicating differences in attribute estimates and their relative importance based on the location of the opt-out option (i.e., either in the first or the second part of the DCE questionnaire). Therefore, location was included as an interaction term with all of the attributes, which resulted in a significant estimate with; out-of-pocket costs, expected outcome, consultation structure and menu schedule. Respondents who answered the choice tasks with opt-out first, showed stronger preferences for out-of-pocket costs and menu schedule and less pronounced preferences for the expected outcome and consultation structure compared to the respondents who first completed the forced-choice tasks.

Finally, attribute estimates were analyzed separately for these two groups (Table 4). Except for the menu schedule attribute, results are highly overlapping. Preference heterogeneity was shown in both models for the expected outcome attribute.

**Table 4:** Attribute estimates (standard errors) of the MIXL stratified by location of the opt-out.

		Opt-out in first part		Opt-out in second part	
Attribute		Beta value	SE	Beta value	SE
Constant	Mean	0.11	0.13	−0.91***	0.25
	Standard deviation	1.93***	0.75	3.31***	0.97
Menu schedule	Flexible	0.12*	0.07	−0.08	0.08
	General (ref)	−0.04	0.03	0.11***	0.00
	Elaborate	−0.07	0.06	−0.02	0.06
Physical activity schedule	Flexible	−0.13**	0.06	−0.14**	0.07
	General (ref)	0.04	0.01	0.01	0.01
	Elaborate	0.09	0.06	0.13**	0.06
Consultation structure	Individual (ref)	0.48***	0.01	0.56***	0.01
	Groups of 5	−0.06	0.06	0.09	0.07
	Groups of 10	−0.42***	0.09	−0.65***	0.10
Expected outcome (10 kg)	Mean	0.61***	0.19	0.87***	0.15
	Standard deviation	1.65***	0.75	2.12***	0.50
Out-of-pocket costs (€100)		−0.71***	0.08	−0.86***	0.09

* significant at p<.10;

**significant at p<.05;

*** significant at p<.001.

### Sensitivity analysis

To obtain more insight into the robustness of the retrieved results, three separate additional analyses were performed. First, respondents who answered inconsistently in more than one choice task (16.6%) were excluded from the analysis. Secondly, respondents were excluded if they indicated (in the first part of the questionnaire) that they would probably not or certainly not participate in a future lifestyle program (53.6%), no matter what attributes this lifestyle program would have. Third, all respondents who chose the opt-out option in all choice tasks were excluded (21.6%). The results of these analyses did not show any notable differences from the analysis described above with respect to the results on the forced choice data and opt-out data (results not shown).

### Willingness to pay

Table 5 shows the different WTP values for the attribute levels that were statistically significant in both the forced-choice and the opt-out model. Based on the forced-choice model, respondents reported a significant higher WTP estimate for a switch from a flexible PA schedule to a more general schedule, compared to the opt-out model (i.e., respectively 44 and 19 euro per year). Within the forced choice model, the WTP for individual consultations instead of consultation in groups with 10 other patients was approximately €114 per year, while in the opt-out model the WTP was estimated to be approximately €134 per year for this same switch in consultation structure. With respect to an expected additional weight loss of 10 kilograms, the forced-choice model showed a WTP estimate of €77 compared to a WTP estimate of approximately €98 for the opt-out model. Though these WTP values differ on an absolute scale, both difference were not statistically significant as the accompanying confidence intervals overlapped.

**Table 5:** Willingness to pay values for all tested models from table 3 and 5.

	Switch from a flexible to a general PA schedule		Switch from consultation in groups of 10 to individual consultation		Every 10 kg of extra anticipated weight loss	
	WTP (€)	95%CI	WTP (€)	95%CI	WTP (€)	95%CI
Forced model	44.3*	29.6; 59.0	113.5	68.4; 158.5	77.2	56.7; 97.8
Opt-out model	19.3*	13.5; 25.0	134.1	100.2; 168.1	98.1	69.8; 126.3
Opt-out in first part of the DCE	23.7	17.1; 30.4	125.8	75.8; 175.9	85.4	32.9; 137.9
Opt-out in second part of the DCE	17.8	8.9; 26.6	140.7	88.5; 192.8	101.2	66.0; 136.4

* Significant at p<.05.

WTP values differed between the data where the opt-out was offered first or seconds, but these differences were not statistically significant due to the large confidence intervals around these estimates (Table 5).

## Discussion

Results show differences in opting out based on the location of the opt-out option and the educational level of the respondents. The attribute estimates between the forced-choice and opt-out dataset differed, but no notable differences in the relative order of the attributes (as compared to each other) were present. However, because the importance weights of the attributes did differ between the datasets, there is a statistically significant difference in the WTP of patients for a PA schedule. This difference could lead to different conclusions and recommendations with regard to developing a lifestyle program that is most attractive for the target group.

Current findings underline the statements of Dahr and colleagues (2003) that the independence of irrelevant alternatives (IIA) assumption for forced-choice data does not hold in unforced data [9]. If this assumption would hold, including an opt-out option would not change study outcomes as it would take equal proportions of all attribute estimates [3], [6], [9]. If this were to be the case, including an opt-out option would not be necessary for the accurate prediction of a patient's' preferences. It is, however, more likely that adding an opt-out option to a forced choice model will disproportionally change study results because this option competes more with one scenario than the other in the same choice task [51]. Current results confirm that this IIA assumption does not hold because a disproportional shift was shown for choosing option A or B in the forced choice sets and then moving to opt-out in the unforced choice sets. Moreover, study results change slightly if an opt-out option is included.

While the direct results of the DCE might not have differed much between the forced and opt-out data, the analysis of the influence of the location of the opt-out option (either in the first nine choice tasks or the second nine choice tasks), showed clearly that the location of the opt-out option in the questionnaire influences the results of a DCE. This was also shown in previous research [13]. The fact that, in general, fewer people chose to opt-out when this was offered in the second part of the questionnaire might be interpreted as a learning effect that respondents go through when completing a DCE. These respondents were forced to make a choice at first, so they became familiar with completing a DCE choice task. Respondents might therefore have had a lower tendency to opt-out when this option was offered later on (unless they really did not want to participate in a lifestyle intervention). These results are in line with findings from previous research, that indicate a decrease in negative emotions and decisions to opt-out when individuals become more familiar with making trade-offs [9], [11]–[13], [16]. It was hypothesized that respondents with a lower education would more often find choice tasks to be complex; this study showed that respondents with a lower educational level significantly more often opted out. This result underlines our hypothesis that respondents more often opt-out if they find the choice sets complex.

Additionally to the effect on the data by simply including such an opt-out option, our study results indicate that choice behavior changes which influences DCE results when respondents are given the opportunity to opt-out. Including an extra choice option automatically implies reduced effectiveness, as there are more answering categories included. Specifically an opt-out option does not provide any insight on attribute level trade-offs. This is not an issue, if the choice to opt-out is due to the low perceived personal utility of the other scenarios. However, our analysis showed that it is likely to assume that a considerable number of respondents chose to opt-out for other reasons than a dislike for lifestyle programs. It can therefore be suggested that including an opt-out option in a DCE, leads to an ‘unnecessary’ loss of effectiveness. This is of special interest in the light of designing DCEs in an efficient manner (e.g., by minimizing D-error). Such designs strive to create choice sets with an optimal utility balance between the scenarios of each choice task, by optimizing the variance-covariance matrix (22–24). This designing procedure results in a DCE that requires a minimal number of choice tasks per respondent and a minimal number of respondents per experiment (aside from model specifications (e.g., level restrictions or interactions)). At the same time, this may induce complexity of the generated choice tasks. Since there are indications for higher levels of opt-out when choice tasks become more complex, the efficiency of designing DCEs in such a way may be at risk. Future research is necessary to identify subgroups among study populations that are most likely to opt-out due to other reasons than solely personal utility. Moreover, it should be explored how DCEs can be designed in an efficient manner while keeping in mind this phenomenon.

The current study has some limitations. Although an efficient DCE design was developed, an even more efficient design could have been created if the beta priors retrieved with the pilot-study were more stable. If more informed beta priors would have been used (instead of using zero as a beta prior for all attributes), the expected preferences of the target population would already be included in the design of the DCE. This way, the design varies the attribute levels based on their relative importance as displayed in the beta priors, resulting in more complex choice tasks. Since choice task complexity is expected to drive the choice to opt-out, using a more efficient design would probably have led to even more individuals that chose to opt-out and thereby more pronounced differences between the results of the model with and without an opt-out option.

The response rate was 31.2%. Due to confidentiality agreements with the care groups that distributed the questionnaires, no reminder letters could be distributed and a non-response analysis could not be conducted. Non-response is likely to be selective, in the sense that DM2 patients who are not interested in a lifestyle program were less likely to participate in this study. It is therefore expected that this selective response resulted in an underestimation of the differences between the datasets with and without an opt-out option. Although the conclusions of this paper would probably not change, they might have been more pronounced if respondents with a negative attribute towards lifestyle programs had participated.

The current study included DM2 patients in the age category 35–65. There is very limited information on the representativeness of the current study population compared to the target population. Additional analysis of DM2 patients aged 35–65 in a large Dutch Cohort study (EPIC-NL [52]) showed the same mean BMI values, It was not possible to compare other characteristics due to specific inclusion criteria of the EPIC-NL study. However, it is expected in the current sample that especially the number of respondents from an ethnic minority (7.3%) is relatively low compared to the average population of DM2 patients aged 35–65 years. If the hypothesized learning effect (i.e., people choose to opt-out because they do not understand the questions in a DCE or if the choice that has to be made is too difficult) is indeed present, it could very well be that the differences in results due to the inclusion of an opt-out option are underestimated assuming that, due to language difficulties, non-Dutch DM2 patients were more likely to opt-out.

## Conclusions

In general, the choice for including an opt-out option in DCEs, depends evidently on the research objective. When the research objective is to determine the potential participation in a health program, an opt-out option should always be included; if in real life ‘not participating’ is an option as well. By doing so, researchers stay as close as possible to the actual choices of their target population. Introducing an additional loss of power, because respondents do not make any trade-offs and chose to opt-out, should then be accepted. However, the number of respondents that opt-out for other reasons than aiming for the highest personal utility should be minimized. Based on the learning effect that was shown in this study, future DCEs that include an opt-out option may want to incorporate multiple forced choice warm-up exercises. However, since DCE questionnaire are already cognitively demanding and time consuming, a more efficient solution might be to use a dual response design. In such a design, respondents are forced to make a choice and immediately after choosing, respondents are asked if they would like to opt out if given the choice [53]–[55]. This might diminish the risk that a direct introduction of an opt-out results in large numbers of respondents avoiding to seriously weigh the different levels of attributes. Additionally, in order to minimize the proportion of respondents that chooses to opt-out because they find the choice tasks too complex or difficult, future research should empirically explore how choice sets should be presented to make them as easy and less complex as possible. Finally, additional research that uses debriefing of respondents should be conducted to explore the reasons for choosing the opt-out alternative in depth.

## References

[1] Lancsar E, Louviere J (2008) Conducting discrete choice experiments to inform healthcare decision making: a user's guide. Pharmacoeconomics 26: 661–677. 18620460 10.2165/00019053-200826080-00004

[2] Ryan M, Farrar S (2000) Using conjoint analysis to elicit preferences for health care. BMJ 320: 1530–1533. 10834905 10.1136/bmj.320.7248.1530 PMC1118112

[3] Ryan M, Gerard K, Amaya-Amaya M (2008) Using Discrete Choice Experiments to Value Health and Health Care; Bateman IJ, editor. Dordrecht: Springer.

[4] de Bekker-Grob EW, Ryan M, Gerard K (2012) Discrete choice experiments in health economics: a review of the literature. Health Econ 21: 145–172. 22223558 10.1002/hec.1697

[5] Cascetta E (2009) Random Utility Theory. In: Cascetta E, editor. Transportation Systems Analysis: models and applications. New York: Springer. pp. 89–167.

[6] Louviere JJ, Hensher DA, Swait JD (2000) Stated Choice Methods; analysis and apllication. Cambridge: Cambridge University Press.

[7] de Bekker-Grob EW, Chorus CG (2013) Random regret-based discrete choice modelling: An application to health care. PharmacoEconomics 31: 623–634. 23620214 10.1007/s40273-013-0059-0

[8] Karni E, Schwartz A (1977) Search theory: the case of search with uncertain recall. J Econ Theory 16: 38–52.

[9] Dhar R, Itamar S (2003) The effect of forced choice on choice. J Market Res 40: 146–160.

[10] Krosnick JA, Holbrook AL, Berent MK, Carson RT, Hanemann WM, et al (2002) The impact of “no opinion” response options on data quality: non-attitude reduction or an invitation to satisfice? Public Opin Q 66: 371–403.

[11] Dhar R (1997) Consumer preference for a no-choice option. J Cons Res 24: 215–231.

[12] Luce MF, Payne JW, Bettman JR (1999) Emotional trade-off difficulty and choice. J Markt Res 36: 143–159.

[13] Boxall P, Adamowicz WL, Moon A (2009) Complexity in choice experiments: choice of status quo alternative and implications for welfare measurement. Auss J Arg Resour Ec 53: 503–519.

[14] Iyengar SS, Kamenica E (2007) Choice overload and simplicity seeking. Working paper

[15] Kahneman D, Knetsch JL, Thaler RH (1991) The endowment effect, loss aversion and status quo bias. J Econ Perspect 5: 193–206.

[16] Luce MF (1998) Choosing to avoid: coping with negative emotion-laden consumer desicions. J Cons Res 24: 409–433.

[17] Meyerhoff J, Liebe U (2009) Status quo effect in choice experiments: emperical evidence on attitude and choice task complexity. Land Econ 85: 515–528.

[18] Nowlis SM, Kahn BE, Dhar R (2002) Coping with ambivalence: the effect of removing a neutral option on consumer attitude and preference Judgements. J ConsRes 29: 319–334.

[19] Ritov I, Baron J (1992) Status quo and ommission biases. Journal Of Risk and Uncertainty 5: 49–61.

[20] Tversky A, Shafir E (1992) Choice under conflict: the dinamics of differed discision. Psychol Sci 3: 358–361.

[21] van Heafen RH, Massey DM, Adamowics WL (2005) Serial nonperticipation in repeated discrete choice models. Am J Agr Ec 87: 1061–1076.

[22] Cook J, Whittington D, Canh AG, Johnson FR, Nyamete A (2007) Reliability of stated preferences for cholera and typhoid vaccines with time to think in Hue, Vietnam. Econ Inq 45: 100–114.

[23] Ciliska D, Thomas H, Buffet C (2012) An Introduction to Evidence-Informed Public Health and A Compendium of Critical Appraisal Tools for Public Health Practice (Revised). Hamilton ON: National Collaborating Centre for Methods and Tools.

[24] Elwyn G, Frosch D, Rollnick S (2009) Dual equipoise shared decision making: definitions for decision and behaviour support interventions. Implement Sci 4. 10.1186/1748-5908-4-75 PMC2784743 19922647

[25] Yousefi-Nooraie R, Dobbins M, Brouwers M, Wakefield P (2012) Information seeking for making evidence-informed decisions: a social network analysis on the staff of a public health department in Canada. BMC Health Serv Res 12. 10.1186/1472-6963-12-118 PMC3496590 22591757

[26] Struijs JN, Baan CA (2011) Integrating care through bundled payments–lessons from The Netherlands. N Engl J Med 364: 990–991. 21410368 10.1056/NEJMp1011849

[27] Dube MC, Valois P, Prud'homme D, Weisnagel SJ, Lavoie C (2006) Physical activity barriers in diabetes: development and validation of a new scale. Diabetes Res Clin Pract 72: 20–27. 16256239 10.1016/j.diabres.2005.08.008

[28] Forbes CC, Plotnikoff RC, Courneya KS, Boule NG (2010) Physical activity preferences and type 2 diabetes: exploring demographic, cognitive, and behavioral differences. Diabetes Educ 36: 801–815. 20736386 10.1177/0145721710378538

[29] Lakerveld J, Ijzelenberg W, van Tulder MW, Hellemans IM, Rauwerda JA, et al (2008) Motives for (not) participating in a lifestyle intervention trial. BMC Med Res Methodol 8: 17. 18402683 10.1186/1471-2288-8-17 PMC2365955

[30] Nagelkerk J, Reick K, Meengs L (2006) Perceived barriers and effective strategies to diabetes self-management. J Adv Nurs 54: 151–158. 16553701 10.1111/j.1365-2648.2006.03799.x

[31] Owen K, Pettman T, Haas M, Viney R, Misan G (2010) Individual preferences for diet and exercise programmes: changes over a lifestyle intervention and their link with outcomes. Public Health Nutr 13: 245–252. 19656436 10.1017/S1368980009990784

[32] Gils van PF, Lambooi MS, Flanderijn MHW, van den Berg M, de Wit GA, et al (2011) Financial incentives and other factors on willingness to participate in a lifestyle intervention program in diabetic patients: a conjoint analysis. Patient Prefer Adherence 5: 537–546. 22114468 10.2147/PPA.S16854 PMC3218115

[33] Roux L, Ubach C, Donaldson C, Ryan M (2004) Valuing the benefits of weight loss programs: an application of the discrete choice experiment. Obes Res 12: 1342–1351. 15340118 10.1038/oby.2004.169

[34] Thomas DE, Elliott EJ, Naughton GA (2006) Exercise for type 2 diabetes mellitus. Cochrane Database Syst Rev 3: CD002968. 10.1002/14651858.CD002968.pub2 PMC8989410 16855995

[35] Thomas N, Alder E, Leese GP (2004) Barriers to physical activity in patients with diabetes. Postgrad Med J 80: 287–291. 15138320 10.1136/pgmj.2003.010553 PMC1742997

[36] Vijan S, Stuart NS, Fitzgerald JT, Ronis DL, Hayward RA, et al (2005) Barriers to following dietary recommendations in Type 2 diabetes. Diabet Med 22: 32–38. 15606688 10.1111/j.1464-5491.2004.01342.x

[37] Wood FG (2002) Ethnic differences in exercise among adults with diabetes. West J Nurs Res 24: 502–515. 12148832 10.1177/019394502400446388

[38] Bliemer MCJ, Rose JM (2009) Efficiency and sample size requirements for stated choice experiments. Transportaion Research Broad Annual Meeting. Washington DC.

[39] Huber J, Zwerina K (1996) The Importance of Utility Balance in Efficient Choice Designs. J Market Res 33: 307–317.

[40] Cheraghi-Sohi S, Bower P, Mead N, McDonalds R, Whalley D, et al (2007) Making sense of patient priorities: applying discrete choice methods in primary care using ‘think aloud’ technique. Family Practice 24: 276–282. 17478438 10.1093/fampra/cmm007

[41] Coast J, Horrocks S (2007) Developing attributes and levels for discrete choice experiments using qualitative methods. J Health Serv Res Policy 12: 25–30. 17244394 10.1258/135581907779497602

[42] Marshall D, Bridges BF, Hauber B, Cameron R, Donnalley L, et al (2010) Conjoint Analysis Applications in Health - How are Studies being Designed and Reported?: An Update on Current Practice in the Published Literature between 2005 and 2008. Patient 3: 249–256. 22273432 10.2165/11539650-000000000-00000

[43] Lamers LM, Stalmeier PF, McDonnell J, Krabbe PF, van Busschbach JJ (2005) [Measuring the quality of life in economic evaluations: the Dutch EQ-5D tariff]. Ned Tijdschr Geneeskd 149: 1574–1578. 16038162

[44] Louviere JJ, Islam T, Wasi N, Street D, Burgess L (2008) Designing discrete choice experiments: do optimal designs come at a price? J Cons Res 35: 360–375.

[45] Bech M, Gyrd-Hansen D (2005) Effects coding in discrete choice experiments. Health Econ 14: 1079–1083. 15852455 10.1002/hec.984

[46] Lancsar E, Louviere J (2006) Deleting ‘irrational’ responses from discrete choice experiments: a case of investigating or imposing preferences? Health Econ 15: 797–811. 16615039 10.1002/hec.1104

[47] Miguel FS, Ryan M, Amaya-Amaya M (2005) ‘Irrational’ stated preferences: a quantitative and qualitative investigation. Health Econ 14: 307–322. 15386664 10.1002/hec.912

[48] Ryan M, Watson V, Entwistle V (2009) Rationalising the ‘irrational’: a think aloud study of discrete choice experiment responses. Health Econ 18: 321–336. 18651601 10.1002/hec.1369

[49] Sen A (1993) Internal Consistency of Choice. Econometrica 61: 495–521.

[50] Revelt D, Train KE (1998) Mixed Logit with Repeated Choices: Households' Choices of Appliance Efficiency Level. Rev Econ Stat 80: 647–657.

[51] Shafir E, Simonson I, Tversky A (1993) Reason based choice. Cognition 49: 11–36. 8287671 10.1016/0010-0277(93)90034-s

[52] Beulens JW, Monninkhof EM, Verschuren WM, van der Schouw YT, Smit J, et al (2010) Cohort profile: the EPIC-NL study. Int J Epidemiol 39: 1170–1178. 19483199 10.1093/ije/dyp217

[53] Brazell JD, Diener CG, Karniouchina E, Moore WL, Severin V, et al (2006) The no-choice option and dual response choice designs. Market Lett 17: 255–268.

[54] Marshall DA, Johnson FR, Kulin NA, Ozdemir S, Walsh JM, et al (2009) How do physician assessments of patient preferences for colorectal cancer screening tests differ from actual preferences? A comparison in Canada and the United States using a stated-choice survey. Health Econ 18: 1420–1439. 19191268 10.1002/hec.1437 PMC3964796

[55] Marshall DA, Johnson FR, Phillips KA, Marshall JK, Thabane L, et al (2007) Measuring patient preferences for colorectal cancer screening using a choice-format survey. Value Health 10: 415–430. 17888107 10.1111/j.1524-4733.2007.00196.x

